# Visitors’ experiences of public and private dental care in Sweden in 1992–2012

**DOI:** 10.1038/s41405-019-0020-1

**Published:** 2019-08-22

**Authors:** Raimo Pälvärinne, Dowen Birkhed, Birger Forsberg, Eeva Widström

**Affiliations:** 1 Department of Oral Diagnostics, Faculty of Odontology, Malmö University, Malmö, Sweden; 2 Professor Emeritus, Malmö, Sweden; 3 Department of Public Health Sciences, Karolinska Institutet, Stockholm, Sweden; 4 Institute of Clinical Dentistry, Arctic University, Tromso, Norway

**Keywords:** Dental epidemiology, Dental treatment planning

## Abstract

**Aim:**

The aim was to compare adult patients’ experiences of public and private dental care in Sweden over time from the ages of 50 and 70 years, between 1992 and 2012.

**Materials and methods:**

Data on visiting patterns, oral health, fees and satisfaction were obtained from a questionnaire study every 5 years in 1992–2012 and analysed by using the Chi-square test and logistic regression. In the present study, the answers given by 6083 respondents in 1992 and 5220 in 2012 were included.

**Results:**

Of the 50-year olds, 73.5% had visited the private sector and 26.5% the public sector. In 1992, patients in the public dental service (PDS) had visited their dentists less frequently and experienced having a slightly poorer dental status compared with private patients. After 20 years (2012), the distribution of patients between the two sectors was almost the same (71.4% and 28.8%) and the differences in visiting pattern and dental health persisted. During the study period, 21.6% of the patients changed treatment sector. A small proportion of patients had high treatment costs. A larger proportion of the private sector visitors than the PDS visitors were consistently satisfied with the dental care they had received.

**Conclusions:**

As a whole, most adult patients in Sweden were satisfied with their dental care at both public and private clinics.

## Introduction

In Sweden, there are both public and private oral health-care providers. The public dental service (PDS, Folktandvården) is operated by all 21 county councils/regions (20 CCs and one municipality), with approximately 880 dental clinics. Private dental care comprises approximately 2000 care providers (many with more than one dentist) with approximately 3550 dental surgeries. Of the 7528 dentists (in 2013), 4070 (54%) were publicly employed and 3458 (46%) worked in the private sector.^1^ The number of dentists has been relatively constant since the beginning of the 1990s, with about 7500 professional dentists, and this also applies to the distribution between the sectors. Approximately 60% of adult patients visit private dental care providers, while 40% visit the PDS.^2^

In 1992, two of the CCs, Örebro (T) and Östergötland (E), started a prospective population study within dentistry. The aim was to ask all 50-year-old residents in these CCs about their opinions of their own teeth and the dental care provided for them. CC politicians initiated the study. The purpose was to study the extent to which the CCs met the legal requirements relating to the availability of dental services when needed by the residents. In addition to providing a basis for planning dental care for groups of elderly people in the CCs, the results were going to be used by the National Board of Health and Welfare in its work on indicators of good dental care.^3^ The study was repeated every 5 years until 2012 and it has resulted in a number of publications on the respondents’ opinions of their oral health, dental care habits and attitudes to and experiences of dental care.^4,5^ Most respondents in 1992 felt that their oral health was good (89%) and 64% said they attended a dental clinic at least once a year. Satisfaction with dental care was high (94%).^6^

To date, the database generated from the surveys in the two counties, T and E, has not been used for a comparison of public and private providers. In other countries, treatment in the public sector is less expensive for the patients than in the private sector and the PDS is more frequently used by people with a lower income and/or lower education than the private sector. Public sector patients may belong to so-called special needs groups and the treatment may differ in comparison with the private sector.^7–9^ We felt it would be interesting to explore whether this was also the case in Sweden in the present study.

In 1992, when the study started, all dental treatment (including bridgework and prosthetics) for adults was generously (25–75%) subsidised by the government. The PDS and the private sector had the same fixed fees and a high-cost protection system gave extra support to patients with large-scale treatment needs.^10^ In 1998, the subsidy system was reformed. In brief, subsidies were restricted to “basic dental care” for all adults (not including examinations, prosthodontics or orthodontics). Subsidies were higher for people with disease and disability. Subsidy for “contract care” was introduced. Establishment control was taken away and free pricing was introduced, enabling the private dentists and the CCs to set the service charges as they chose.^11^ This resulted in private dental care becoming more expensive than public care.^12^

The next dental reform was introduced in 2008. The system established then is still in place today in 2019. An annual “general dental allowance” (Allmänna TandvårdsBidraget, ATB) was introduced for all adults; it was 150 or 300 SEK (£14 or 28), depending on age. A “protection against high costs” with a subsidy from the government was also introduced. This cap on private spending was based on reference prices defined by the government, as providers were free to set patient fees.^13^ This means that, in comparison with 1992 and 1997, adults had to cover a considerably larger share of their dental costs out of pocket in 2002–2012. On the other hand, adults’ oral health has improved during that period. According to a longitudinal study in the City of Jönköping in Southern Sweden: *“*The proportions of edentulous individuals aged 40–70 years were 16%, 12%, 8%, 1% and 0.3% in 1973, 1983, 1993, 2003 and 2013, respectively. No complete denture wearer younger than 80 years was found in 2013. During the 40-year period, the mean number of teeth in the 30- to 80-year age groups increased. In 2013, the 60-year olds had almost complete dentitions”.^14,15^

## Aim

The aim of this study was to investigate adults’ experiences and opinions of the dental care they have received over time from the age of 50–70 years. Patients’ dental visiting patterns, satisfaction with care, oral health measured as the numbers of teeth and fees paid are compared between the two types of provider, public and private. We also sought to explore possible changes in the use of the two provider groups during the study period and differences between the patients who had visited the public sector and those who had visited the private sector at the start and the end of the study. In addition, a longitudinal follow-up was conducted among those who claimed to have visited only the public sector or the private sector and those who claimed to have used both sectors during the whole study period.

## Material and methods

The data in this study emerged from a data collection procedure at the beginning of 1992, when all 50-year-old residents (*n* = 8888) in the Counties of Örebro (T) and Östergötland (E) were sent a postal questionnaire relating to their “experiences of dental care and oral health”. The questionnaire was validated and then approved by the Ethical Review Board in Uppsala (Dnr 2011/336). Basically the same questionnaire was sent to the cohort born in 1942 every 5 years until 2012. In 1992, the response rate was 71.4% (*n* = 6343), in 1997, 74.3% (*n* = 6513), in 2002, 75.0% (*n* = 6372), in 2007, 73.1% (*n* = 6078) and, in 2012, 72.2% (*n* = 5697).^6^ In the present study, the answers were analysed from 6083 respondents in 1992 and 5220 respondents in 2012, who stated that they had visited the PDS or private clinics.

This study focused on the following variables: (a) frequency of dental visits (How often did you make dental visits during the last 5 years?), (b) treatment sector (Where did you mainly have dental care during the last 5 years?), (c) cost of dental treatment paid by the patients (How much did you pay out of pocket for dental care during the last year?), (d) satisfaction with the treatment (Are you generally satisfied/not satisfied with the dental care you have received?) and (e) the number of their own teeth remaining (How many of your own teeth do you have?). The alternative answers to all the questions are shown in Table 1. Background factors were education, gender, marital status and country of birth. All respondents did not always answer all the questions.

**Table 1:** Comparison of the 50-year-old respondents’ dental visiting patterns, dental health (numbers of teeth), cost of dental care during the last year, satisfaction with care received, gender, education and marital status by treatment sector used (the PDS or private) at the latest dental visit in 1992 (or before)

	PDS	Private	Total	*p*-Value
*n*	1612	4471	6083	
Frequency of visits during the last 5-year period, *n* (%)				<0.001
Every 2nd year or more seldom	274 (17.0)	208 (4.7)	482 (7.9)	
Once a year	1039 (64.5)	2961 (66.2)	4000 (65.8)	
Twice a year or more often	296 (18.4)	1297 (29.0)	1593 (26.2)	
Missing	3 (0.2)	5 (0.1)	8 (0.1)	
Number of teeth, *n* (%)				<0.001
Edentulous or very few teeth left	76 (4.7)	70 (1.6)	146 (2.4)	
Missing rather a lot of teeth	377 (23.4)	693 (15.5)	1070 (17.6)	
Missing a single tooth	855 (53.0)	2518 (56.3)	3373 (55.4)	
All teeth left	276 (17.1)	1081 (24.2)	1357 (22.3)	
Missing	28 (1.7)	109 (2.4)	137 (2.3)	
Cost of care paid by the patient during the last year, *n* (%)				<0.001
Nothing	115 (7.1)	138 (3.1)	253 (4.2)	
Less than 300 SEK (£25.1)	400 (24.8)	1195 (26.7)	1595 (26.2)	
301–1000 SEK (£25.2–83.8)	727 (45.1)	2097 (46.9)	2824 (46.4)	
More than 1000 SEK (£83.8)	295 (18.3)	946 (21.2)	1241 (20.4)	
Missing	75 (4.7)	95 (2.1)	170 (2.8)	
Satisfaction with care received, *n* (%)				<0.001
Fairly or very dissatisfied	111 (6.9)	202 (4.5)	313 (5.1)	
Generally satisfied	792 (49.1)	1845 (41.3)	2637 (43.4)	
Very satisfied	681 (42.2)	2366 (52.9)	3047 (50.1)	
Missing	28 (1.7)	58 (1.3)	86 (1.4)	
Gender = male, *n* (%)	815 (50.6)	2197 (49.1)	3012 (49.5)	0.338
Level of education, *n* (%)				<0.001
No university degree	1288 (79.9)	3487 (78.0)	4775 (78.5)	
University degree	300 (18.6)	959 (21.4)	1259 (20.7)	
Missing	24 (1.5)	25 (0.6)	49 (0.8)	
Marital status, *n* (%)				0.098
Single	310 (19.2)	797 (17.8)	1107 (18.2)	
Married/cohabiting	1297 (80.5)	3669 (82.1)	4966 (81.6)	
Missing	5 (0.3)	5 (0.1)	10 (0.2)	
Country of birth, *n* (%)				<0.001
Sweden	1455 (90.3)	4248 (95.0)	5703 (93.8)	
Other Nordic country	72 (4.5)	102 (2.3)	174 (2.9)	
Other country	85 (5.3)	120 (2.7)	205 (3.4)	
Missing	0 (0.0)	1 (0.0)	1 (0.0)	

*N* PDS = 1612 persons and *N* private sector visitors = 4471 persons. *N*-values fluctuate because all respondents did not answer all the questions

### Statistical methods

Chi-square tests were performed on differences between the PDS and private visitor cohorts at baseline and in 2012. The Chi-square test was also used to analyse changes in visiting patterns, number of teeth and satisfaction between 1992 and 2012 for patients who never changed dental care provider sector during the 20-year period. In order to further analyse the factors that might influence the odds of having “all teeth left” after a 20-year follow-up, a logistic regression was performed. *p*-Values below 0.05 were considered to be significant.

Statistical analyses were conducted using R version 3.4.2 for Windows.^16^

## Results

### Adults 50 years of age in 1992

In 1992, most of the then 50-year-old respondents (4471; 73.5%) had visited the private sector and 1612 (26.5%) the public sector. There were no statistically significant differences between the private and public visitors as regards gender or marital status, but PDS visitors had a significantly lower educational level (*p* < 0.05) and a larger proportion of them were born outside Sweden (*p* < 0.001; Table 1). Respondents who had made their latest dental visits to the PDS had fewer of their own teeth than those who had visited private dentists; e.g. 17.4% of the former had all their teeth left in comparison to 24.2% of the latter (*p* < 0.001; Table 1). The public visitors had visited their dental clinic more seldom than the private visitors, they were less satisfied with the care they had received (*p* < 0.001) and they had paid less for their treatment (*p* < 0.05; Table 1). Of all the respondents, 76.8% claimed to have paid less than 1000 SEK (£83.4), corresponding to 1422 SEK (£119.2) in today’s monetary value.^17^ In 1992, the average income for 50-year-old Swedes was 15604 SEK (£1271.4).^18^

### Adults 70 years of age in 2012

In 2012, the total number of respondents had fallen from 6083 in 1992 to 5220 (8.7%). The proportion of respondents who had most recently visited the private sector was 71.4% (3738) and, of those having visited the public sector, 28.8% (1482). These proportions were almost at the same level as 20 years earlier. The proportion of private visitors with a university education (27.6%) was significantly higher (*p* < 0.001) than that of the public visitors, 22.3% (Table 2). The respondents as a whole had a higher educational level in 2012 than in 1992. According to the answers to the questionnaire, those respondents who had mainly visited the PDS during the past 5 years had, as in 1992, statistically significantly fewer of their own teeth than those who had visited private dentists (*p* < 0.001; Table 2). The public visitors had visited their dental clinic less frequently than the private visitors and they were less satisfied with the care they had received (*p* < 0.001). There were statistically significant differences in costs paid, as the PDS visitors had paid less than the private sector visitors. Of all the respondents, 89.7% claimed to have paid less than 8000 SEK (£670.5), corresponding to 8363 SEK (£701.1) in today’s monetary value (Table 2). In 2012, the average income per month for 70-year-old Swedes was 18,167 SEK (£1480.2).^19^ The general retirement age in Sweden is 65 years.

**Table 2:** Comparison of the 70-year-old respondents’ dental visiting patterns, dental health (numbers of teeth), cost of dental care during the last year, satisfaction with care received, gender, education and marital status by treatment sector used (the PDS or private) at the latest dental visit in 2012 (or before)

	PDS	Private	Total	*p*-Value
*n*	1482	3738	5220	
Frequency of visits during the last 5-year period, *n* (%)				<0.001
Every 2nd year or more seldom	370 (25.0)	218 (5.8)	588 (11.3)	
Once a year	746 (50.3)	2432 (65.1)	3178 (60.9)	
Twice a year or more often	352 (23.8)	1072 (28.7)	1424 (27.3)	
Missing	14 (0.9)	16 (0.4)	30 (0.6)	
Number of teeth, *n* (%)				<0.001
Edentulous or very few teeth left	89 (6.0)	106 (2.8)	195 (3.7)	
Missing rather a lot of teeth	392 (26.5)	730 (19.5)	1122 (21.5)	
Missing a single tooth	817 (55.1)	2284 (61.1)	3101 (59.4)	
All teeth left	141 (9.5)	530 (14.2)	671 (12.9)	
Missing	43 (2.9)	88 (2.4)	131 (2.5)	
Cost of care paid by the patient during the last year, *n* (%)				<0.001
Nothing	164 (11.1)	143 (3.8)	307 (5.9)	
1–2000 SEK (£1–167.6)	745 (50.3)	2163 (57.9)	2908 (55.7)	
2001–8000 SEK (£167.7–670.5)	400 (27.0)	1065 (28.5)	1465 (28.1)	
More than 8000 SEK (£670.5)	79 (5.3)	227 (6.1)	306 (5.9)	
Missing	94 (6.3)	140 (3.7)	234 (4.5)	
Satisfaction with care received, *n* (%)				<0.001
Fairly or very dissatisfied	121 (8.2)	136 (3.6)	257 (4.9)	
Generally satisfied	864 (58.3)	1637 (43.8)	2501 (47.9)	
Very satisfied	485 (32.7)	1949 (52.1)	2434 (46.6)	
Missing	12 (0.8)	16 (0.4)	28 (0.5)	
Gender = male, *n* (%)	748 (50.5)	1828 (48.9)	2576 (49.3)	0.311
Level of education, *n* (%)				<0.001
No university degree	1143 (77.1)	2687 (71.9)	3830 (73.4)	
University degree	330 (22.3)	1031 (27.6)	1361 (26.1)	
Missing	9 (0.6)	20 (0.5)	29 (0.6)	
Marital status, *n* (%)				<0.05
Single	379 (25.6)	830 (22.2)	1209 (23.2)	
Married/cohabiting	1049 (70.8)	2791 (74.7)	3840 (73.6)	
Missing	54 (3.6)	117 (3.1)	171 (3.3)	
Country of birth, *n* (%)				<0.05
Sweden	1370 (92.4)	3548 (94.9)	4918 (94.2)	
Other Nordic country	46 (3.1)	86 (2.3)	132 (2.5)	
Other country	62 (4.2)	97 (2.6)	159 (3.0)	
Missing	4 (0.3)	7 (0.2)	11 (0.2)	

*N* PDS = 1482 persons and *N* private sector visitors = 3738 persons. *N*-values fluctuate because all respondents did not answer all the questions

### Private or public dental care only, or both in 1992–2012

**Fig. 1:**
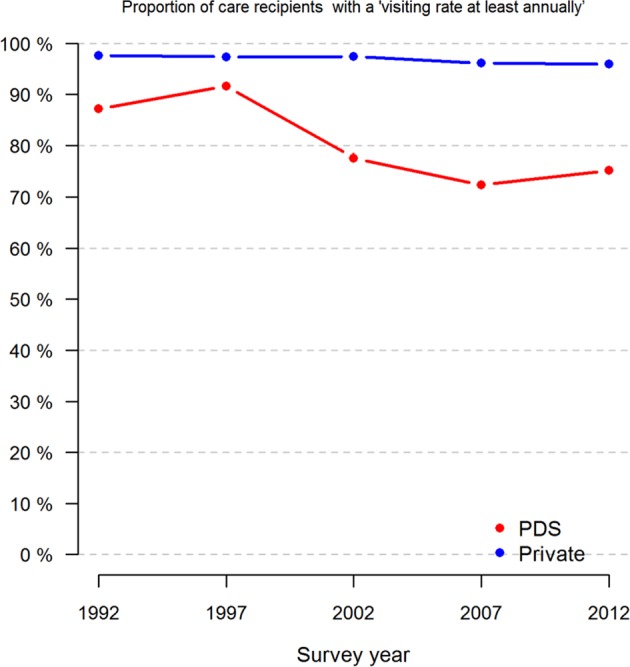
Proportions (%) of respondents who claimed to have made dental visits at least annually (once a year or twice a year or more often) by survey year and treatment sector (PDS or private only)

Of the initial 4471 private visitors and, of the initial 1612 PDS visitors, who answered the questionnaire in 1992 and 2012, 2478 private visitors (55.4%) and 649 public visitors (40.2%) claimed to have visited the same treatment sector all the time. Most private patients (64.8%) claimed to have maintained the same visiting frequency as in 1992 in contrast to 51.0% of the PDS visitors (*p* < 0.001; Table 3, Fig. 1). A statistically significantly larger proportion (25.9%) of the PDS visitors visited their dentist more seldom than before, in contrast to 17.6% of the private visitors (*p* < 0.001; Table 3).

**Table 3:** Changes in visiting patterns, numbers of teeth and satisfaction with care received during the 20 years from 1992 to 2012 by treatment sector

	Mixed	PDS	Private	Total	*p*-Value
*n*	861	649	2478	3988	
Change in visiting pattern from 1992 to 2012, *n* (%)					<0.001
Lower frequency in 2012	214 (24.9)	168 (25.9)	435 (17.6)	817 (20.5)	
Same frequency	467 (54.2)	331 (51.0)	1605 (64.8)	2403 (60.3)	
Higher frequency	174 (20.2)	142 (21.9)	432 (17.4)	748 (18.8)	
Missing	6 (0.7)	8 (1.2)	6 (0.2)	20 (0.5)	
Changes in number of teeth from 1992 to 2012, *n* (%)					0.098
Fewer teeth in 2012	259 (30.1)	158 (24.3)	646 (26.1)	1063 (26.7)	
Same number of teeth	507 (58.9)	418 (64.4)	1539 (62.1)	2464 (61.8)	
More teeth in 2012	53 (6.2)	49 (7.6)	190 (7.7)	292 (7.3)	
Missing	42 (4.9)	24 (3.7)	103 (4.2)	169 (4.2)	
Change in satisfaction from 1992 to 2012, *n* (%)					<0.05
Less satisfied in 2012	206 (23.9)	164 (25.3)	513 (20.7)	883 (22.1)	
Satisfied at the same level	479 (55.6)	378 (58.2)	1480 (59.7)	2337 (58.6)	
More satisfied in 2012	153 (17.8)	98 (15.1)	449 (18.1)	700 (17.6)	
Missing	23 (2.7)	9 (1.4)	36 (1.5)	68 (1.7)	

Respondents who changed treatment sector during the study period are included in the mixed group. The PDS and private groups include those who visited only one sector but may have missed answering some year between 1992 and 2012. *p*-Values are based on those who actively responded (i.e. missing is not included in the calculation)

**Fig. 2:**
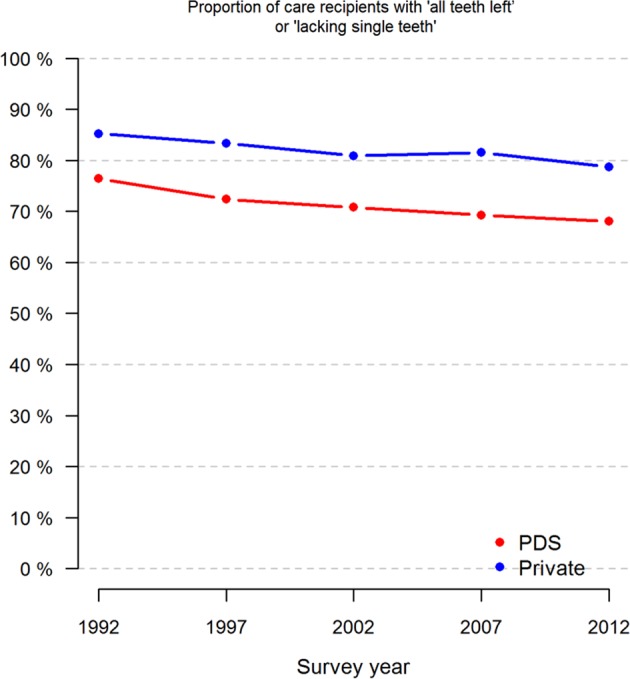
Proportions (%) of respondents who claimed to have kept practically all their teeth by survey year and treatment sector (PDS or private only)

Over time, the proportion of respondents who claimed to have retained practically all their teeth decreased in both sectors (Fig. 2). Of the PDS visitors, 64.4% and, of the private sector visitors, 62.1% said that they belonged to the same “numbers of teeth category” during the whole study period (*p* < 0.05). About a quarter (24.3%/26.1%) claimed to have fewer teeth and, interestingly, 7.6%/7.7% had more teeth than initially (Table 3).

**Fig. 3:**
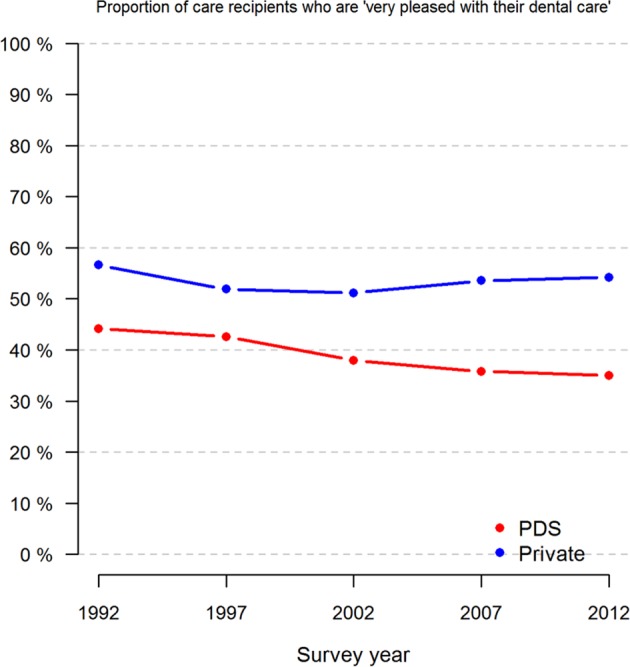
Proportions (%) of respondents who claimed to be very or rather satisfied with the dental care they had by survey year and treatment sector (PDS or private only)

A larger proportion of the private sector visitors than PDS visitors were consistently more satisfied with the dental care they had received (Fig. 3, Table 3). At baseline, there was a difference of 10.7% among “very pleased” in favour of private dentistry. Over time, the private sector appeared to retain this position, while the PDS decreased slightly (Fig. 3).

There was also a group we called “mixed”. Individuals belonging to this group changed their care provision sector during the period. A larger proportion of respondents in this group had “fewer teeth in 2012” than respondents in the PDS and private groups (*p* < 0.05). Their visiting frequency was midway between the PDS and private visitors, as was their satisfaction level (Table 3).

A separate analysis of those who, after the 20-year follow-up period, had “all their teeth left or were only missing a single tooth” (Table 4) showed that university-educated subjects had 45.8% higher odds and private sector visitors 29.6% higher odds of having all their teeth left. Married people had 19.2% higher odds than unmarried people and men had 14.3% higher odds than women of retaining their natural teeth. The results also showed that individuals born outside Sweden had lower odds, 14.8%/16.5%, of retaining all or most of their teeth than native Swedes (Table 4).

**Table 4:** Odds ratios from logistic regression analysis for likelihood at the end of the study period of having “all teeth left, or missing a single tooth”

	Estimate	Std. error	*z*-Value	*p*-Value	OR	Lower	Upper
(Intercept)	1.402	0.210	6.677	0.000	4.064	2.716	6.192
University degree at baseline	0.377	0.159	2.368	0.018	1.458	1.074	2.006
Private dental care at baseline	0.259	0.161	1.612	0.107	1.296	0.939	1.766
Male	0.134	0.133	1.004	0.315	1.143	0.881	1.485
Married/cohabiting	0.176	0.167	1.053	0.292	1.192	0.853	1.643
Born in the Nordic countries	−0.180	0.548	−0.329	0.742	0.835	0.317	2.874
Born outside the Nordic countries	−0.160	0.490	−0.326	0.744	0.852	0.355	2.527

Only respondents who never changed care provider and answered “all teeth left or missing a single tooth” included at baseline. *N* PDS = 393 persons and *N* private = 1705. Adjusted model (all variables included in the same model)

## Discussion

Self-report surveys have been frequently used in Sweden when studying the use of various public services by adults, as the method has been regarded as feasible and reliable, with a response rate that is sufficiently high to generate results with good validity.^20^ The material used in the present study has been found to be representative of the birth cohort born in 1942. It thus provides information on the dental care of 50- to 70-year-old individuals in the two counties surveyed.^21^ The respondents in longitudinal surveys are exposed to a changing societal environment over time and, in this study, also changes in the care provision and financing systems, which may influence their opinions. They also became older, another circumstance that may influence their perspectives.^3^ This must be considered when interpreting the results.

The study showed that most (4471, 73.5%) of the 6083 50-year olds participating in the study in 1992 had made their latest dental visit to the private sector. Was this because of old habits or social segregation? Because the patient fees at that time were fixed and the same in both sectors, one of the most usual reasons in other countries for using the public sector, namely lower fees, did not apply here. Moreover, because there were no formal restrictions for adults to use the PDS, an explanation of the low use of the PDS might be that it was regarded as a care-giver primarily for children and young people. Further, in 2012, 71.6% of the respondents said that their latest visit had been to the private sector, although the free pricing, introduced in 1998, had made treatment in the PDS less expensive than in the private sector.^12^ The private sector is known to be efficient in recalling its patients and regular attenders are more comfortable with dental visits.^22,23^ Our earlier study showed that the Chief Dental Officers (CDOs), who were PDS leaders in the CCs, felt that keeping their former child patients in the PDS when they become adults was an important strategy.^24^ This is supported by the new contract care payment system in Sweden (so-called “Frisktandvård”), allowing a certain annual fee to be paid by the patient irrespective of treatment needed and provided, being most suitable for young adults.^25^

At the beginning of the study, patients who visited the PDS had a slightly poorer dental status, compared with private patients. This is in accordance with studies from other countries.^7,8^ This difference also persisted over the entire examination period (1992–2012). For example, both examined groups experienced a decrease in the group answering “all teeth left”, but the decrease was larger in the PDS, from 17.1% to 9.5%, than in the private sector, from 24.2% to 14.2%. On the other hand, when looking at the variables “lower number of teeth” and “same number of teeth”, the figures showed that the tooth losses are on the same level in both sectors (*p* > 0.05) (Table 3). Because the formulation of the questions did not make it possible to follow the exact numbers of teeth during the study period, differences of this kind are difficult to explain. However, it was obvious that, during the 20-year study period, many of the “ageing” patients lost teeth independent of treatment sector. Those who did not lose teeth were highly educated, visited the private sector and were more often men and married than women or unmarried, which is in accordance with findings in other studies.^26^ Well-educated people are usually able to manage good oral self-care. Private dental clients are able to visit a dentist or a dental hygienist frequently and presumably have frequent opportunities to obtain professional preventive treatment and advice. One explanation of why married people have higher odds than singles of retaining all their teeth might be that married people live a more stable life with fixed routines. It is more difficult to explain why men had higher odds. The difference between men and women was fairly small (14.3%).

One interesting finding in the present study was that about 10% of the respondents claimed that the number of teeth increased during the 20-year period. It may be difficult for patients to differentiate between natural teeth and bridgework and implants and so they may answer that they have more teeth. In Sweden, having decent-looking teeth, your own or artificial, has long been regarded as politically important and prosthetic treatment has been easily available and highly subsidised.^9,11^

An analysis of the visiting frequency revealed that, in the PDS, the pattern of rare visits increased over time and frequent visitors also increased. In the private sector, the visiting frequencies hardly changed at all. This might be due to the practice in private dental care with “a yearly visit to the dentist”, while the PDS has differentiated the recall intervals by risk assessments based on differences in patients’ oral health but probably also influenced by the availability of staff. In Sweden, the PDS has had access problems, depending on a lack of dentists, all over the country and especially in rural areas since 2007.^27^ This may explain the increase in the number of rare visitors. The increase in frequent visitors may be explained by the increase in the number of dental hygienists in the PDS. Dental hygienists have been seen to make an important contribution to dental care and hence the ratio of dental hygienists to dentists (2 per 5 dentists) is by international standards relatively high in Sweden.

Costs paid by the patients were difficult to compare over time, because of the many changes in the state subsidies during the 20-year period and changes to the question of patient costs in the questionnaire. At the start, in 1992, 76.8% of the respondents claimed to have paid very little, less than 1422 in current SEKs (£120) for all their dental treatment during the period of a year. Dental care was generously remunerated by the national dental insurance, as explained in the “Introduction”. Only 18.3% of PDS and 21.2% of private patients paid more than 1422 SEK (£119.2) (*p* < 0.001). In 2012, free pricing had been introduced and the subsidy for dental costs started at 3000 SEK. The price level was much higher, too. In 2012, 89.7% of all patients paid below the high-level limit, which was set at 8363 SEK (£701.0) in today’s monetary value. The number of patients paying more than 8363 SEK was 5.3% in the PDS and 6.1% in the private sector (*p* < 0.001). This indicates that there were very few patients paying high costs, but there were significantly more private patients doing so.

Ståhlnacke et al.^28,29^ noted in their studies that adults’ satisfaction with dental services was high both in general and with the most recent dental visit, where non-visitors within the last year were more dissatisfied than those who had paid a visit during the last year. Having a high cost for care also increased dissatisfaction but to a smaller degree. The authors were not able to document any correlation between socioeconomic factors and service satisfaction.

Hancock et al.^30^ in the UK investigated private or NHS general dental service care and patients’ satisfaction in certain respects. They noted that satisfaction was greater in private dental care due to perceived access and availability and not because of technical skills.

In our study, the satisfaction rate among the private patients stayed high over the years (55% were very satisfied) but decreased for the PDS patients from 45% to 35% (Fig. 3). One possible explanation is that access and availability were greater in private dentistry. Moreover, the treatment profile of the private dentists differs from that of the PDS, which is shown in other investigations.^9^ More advanced and expensive care is provided in the private sector and simpler, less expensive care, like extractions and fillings, in the PDS.^31^ In the above-mentioned study in Finland, adult patients in the PDS were shown to have more examinations and emergency care, while private patients received more comprehensive care.^9^

Finally, there was a group of respondents using both sectors. This mixed group represented 21.6% of the cohort, showing that many people wanted or needed to change their dental treatment supplier over time. People belonging to this mixed group were shown to lose more teeth than the PDS and private sector attenders and to make more frequent dental visits than the PDS visitors but less often than the private sector visitors. They did not change their degree of satisfaction during this period. These sector users appeared to be more irregular attenders and probably visited dentists when needed, due to pain or lost fillings, and want to choose their visits on their own, not via a recall system. Some private and mixed sector visitors may also have needed specialist care, which is predominantly provided in the public sector in Sweden. According to the Swedish National Board of Health and Welfare, 66% of men and 73% of women made dental visits to obtain a basic examination during the 3-year period, from 2015 to 2017. Older people used dental services more often than younger ones.^11^

## Conclusions

There are some differences between PDS and private dentistry in a longitudinal study spanning 20 years. The differences mainly relate to satisfaction, access and availability of dental care. Most private patients appear to visit their dental clinic with the same frequency, once a year or more often, over time, while the PDS has a more uneven visiting pattern. Over 20% of the patients changed provider between the two sectors during a 20-year period. This is an interesting observation, but the reason is not explained in this study.

In Sweden, the regular use of dental services throughout life is regarded as important and is believed to contribute to a good dental appearance and functioning dentition. The two treatment sectors, the PDS and the private sector, play slightly different roles and, as competitors, for example, it is hoped that they will drive quality development forward and display the best possible ability to meet the demands of patients and politicians. It seems apparent from the present study that the two sectors also complement one another.
